# Vitreous Substitutes: Old and New Materials in Vitreoretinal Surgery

**DOI:** 10.1155/2017/3172138

**Published:** 2017-07-13

**Authors:** Camilla Alovisi, Claudio Panico, Ugo de Sanctis, Chiara M. Eandi

**Affiliations:** ^1^Eye Trauma Center, Department of Ophthalmology, Turin Eye Hospital, Turin, Italy; ^2^Department of Surgical Sciences, Eye Clinic, University of Turin, Turin, Italy

## Abstract

Recent developments in vitreoretinal surgery have increased the need for suitable vitreous substitutes. A successful substitute should maintain all the physical and biochemical properties of the original vitreous, be easy to manipulate, and be long lasting. Substitutes can be gaseous or liquid, both of which have associated advantages and disadvantages related to their physical properties and use. Furthermore, new surgical techniques with smaller vitreoretinal instruments have driven the use of more viscous substitutes. In this review, we analyze and discuss the most frequently used vitreous substitutes and look ahead to future alternatives. We classify these compounds based on their composition and structure, discuss their clinical use with respect to their associated advantages and disadvantages, and analyze how new vitreoretinal surgical techniques have modified their use.

## 1. Introduction

The vitreous body is a clear gel that fills the space between the lens and the retina. It constitutes approximately 80% of the volume of the entire eye [1]. In the recent years, the development of new surgical techniques and intravitreous-release drugs has led to a need for improved vitreous substitutes. The ideal vitreous substitute has all the qualities of the vitreous body (transparency, biocompatibility, volume retention, elasticity, and durability) and lacks negative characteristics such as ageing liquefaction and biodegradation. Intensive research is underway to develop new products that resemble the vitreous as closely as possible. In this review, we describe the positive and negative aspects of current and experimental substitutes and evaluate their use in new surgical techniques to repair retinal detachment.

## 2. Method of Literature Search

For this review, a literature search was conducted that utilized Medline, Premedline, EMBASE, SCOPUS, and Cochrane. Papers from 1950 onwards were included on vitreous structure and function while review articles from 2011 onwards were included on vitreous substitutes. The following search terms were used: vitreous substitutes, vitreous humour, vitreous body, ideal vitreous substitutes, tamponade in retinal detachment, gas tamponade in retinal detachment, pneumatic retinopexy, silicone oil in retinal detachment, heavy silicone oil in retinal detachment, hydrogel, hydrogel in retinal detachment, microincision vitreoretinal surgery, and vitreous substitutes. References present in relevant articles were used. Only articles in English were considered.

## 3. Vitreous

### 3.1. Anatomy

Each eye contains approximately 4 mL of vitreous, a transparent gel-like structure. The vitreous can be subdivided into three anatomical regions: the vitreous core, the vitreous base, and the vitreous cortex. The vitreous cortex is the part closest to the retina; it exhibits a variable thickness and a lamellar structure. It contains hyalocytes and densely packed collagen, similar to the vitreous base which covers the ora serrata [2]. The vitreoretinal interface is composed of the internal limiting membrane (ILM), the innermost part of the retina, and the posterior vitreous. The ILM forms the basement membrane of the Müller cells. It consists of type IV collagen which is associated with glycoproteins and contributes to vitreoretinal adhesion and type XVIII which binds opticin. Opticin is a class III small leucine-rich repeat protein which binds to heparin sulfate contributing to vitreoretinal adhesion [3]. The strength of vitreous attachment to the surrounding tissue, including lens, differs according to location. The vitreous is known to be most firmly attached at the vitreous base, the optic nerve and macula, and over retinal vessels.

The vitreous body is routed by the Cloquet's canal, a remnant of the hyaloid artery, which arises from the Martegiani's space at the optic disc to the retrolental space known as the Berger's space. Unlike the vitreous base and cortex, the vitreous core does not contain hyalocytes and is usually the area sampled in proteomic studies because it is the simplest to acquire. All vitreous structures undergo characteristic changes with ageing, including progressive liquefaction and, in some cases, posterior vitreous detachment.

### 3.2. Chemistry

The vitreous is composed of over 98% water. Between 15% and 20% of water is bound to proteins and glycosaminoglycans. Primate studies have demonstrated that the remaining portion is free [4].

#### 3.2.1. Proteins

The average vitreous concentration of proteins is 1200 *μ*g/mL. Albumins (40%) and immunoglobulins are the most prevalent. Iron-binding proteins such as transferrin are synthesized in the vitreous itself and have a protective role in the event of small vitreous haemorrhages via the prevention of iron toxicity [5].

Collagens are insoluble proteins of the vitreous. They form a three-dimensional meshwork within the vitreous gel. Collagen types II, IV, V/XI, VI, and IX are present, with collagen type II to be the most prevalent (65%), followed by type IX (25%), type V/XI (10%), and type IV (<10%) [6]. In vitro studies have demonstrated that Müller cells are able to synthesize collagens, and therefore these cells are thought to generate the vitreous collagens [7].

#### 3.2.2. Glycosaminoglycans

Glycosaminoglycans (GAGs) are important constituents of the vitreous. They are extracellular matrix polysaccharides that contain repeating disaccharide units. Three major groups of GAGs are present: hyaluronic acid (HA), chondroitin sulfate, and heparan sulfate.

*(1) Hyaluronic acid*. Hyaluronic acid (also called hyaluronan) is unique among the GAGs in that it does not contain sulfate and it is not found covalently attached to proteins forming a proteoglycan. Hyaluronic acid polymers are very large (molecular weights of 100,000–10,000,000 DA) and can displace a large volume of water. The immense size of these molecules makes them excellent lubricators and shock absorbers within the joints and the vitreous. The ageing process creates two structural changes: depolymerization of HA and the subsequent loss of collagen IX. The absence of collagen IX induces aggregation of collagen II fibrils (syneresis) and formation of fluid-filled lacunae (synchisis) [8].

*(2) Chondroitin sulfate*. Chondroitin sulfate is a sulfated GAG composed of a chain of alternating sugars (N-acetylgalactosamine and glucuronic acid). It is usually attached to proteins as part of a proteoglycan. In the vitreous, it appears in the form of two proteoglycans: versican and type IX collagen [9]. It is important in maintaining the structural integrity of the tissue and provides resistance against compression.

*(3) Heparan sulfate*. Heparan sulfate has been identified in small amounts in the vitreous, and its role is to maintain adequate spacing between the collagen fibrils. It may also facilitate the regulation of a wide variety of biological processes including development, angiogenesis, and blood coagulation [10], as well as maintaining vitreoretinal adhesion in collaboration with opticin.

#### 3.2.3. Ascorbic acid and cells

Ascorbic acid is found in higher concentrations in the vitreous body than in the plasma [11]. It has an important role in the process of ageing liquefaction and can inhibit neovascularization, as well as increase the proliferation of hyalocytes. Recent studies have shown that the antioxidant properties of ascorbic acid can also reduce early cataract formation [12]. Vitreous body contains three types of cellular element: hyalocytes, fibroblasts/fibrocytes, and macrophages. Fibroblasts and hyalocytes are present on the vitreous surface, and they are also involved in the phagocytosis and/or secretion of collagen.

### 3.3. Physical Properties

The vitreous is not completely homogeneous, an effect resulting from the presence of so many components. The viscoelastic properties of the vitreous result from the interaction between long collagen fibrils and HA. It is well known that numerous ions in the interior travel from the dense and viscous portions (the vitreous base) to the anterior part. This can affect the release and dissemination of intravitreous drugs.

Although the vitreous has not aroused scientific interest for many years, it has numerous and important roles, with the key functions being to (1) sustain the growth, volume, and elasticity of the eye (structural function); (2) maintain transparency and improve the accommodation (optical function); (3) create a barrier to biochemical substances (barrier function); and (4) provide substances for nutrition and metabolism (nutritional function).

#### 3.3.1. Structural function

Recent studies have shown that the growth of the vitreous may modify the growth of the retinal pigment epithelium (RPE) through the production of HA [13]. The vitreous protects the retina and the other structures from the low-frequency mechanical stress, friction, and vibration that are both common and constant in everyday life. Its viscoelasticity acts as an important shock absorber against physical impact, providing more than a space-filling function, particularly in younger people.

#### 3.3.2. Optical function

The most important role of the vitreous is to maintain transparency, enabling the passage of light rays toward the retina. It transmits visible and near-infrared light, in a similar manner to the aqueous [14]. Little light scattering occurs in the vitreous due to the large HA molecules that separate collagen fibers. By supporting the lens capsule, it also aids the accommodation process.

#### 3.3.3. Barrier function

The vitreous can protect the eye, acting as a barrier to various biochemical substances and cells. In this manner, it helps prevent bacterial infection, although it can act as a growth surface for some viral agents. In the healthy eye, the vitreous is an important part of the blood-ocular barrier, inhibiting neovascularization and inflammation.

#### 3.3.4. Nutritional function

Other important functions of the vitreous body are metabolism and the regulation of intraocular oxygen through ascorbate concentration [12]. Consuming oxygen via an ascorbate-dependent mechanism protects the lens from oxidative damage and reduces cataract formation.

## 4. The Ideal Vitreous Substitute

The ideal vitreous substitute is similar to the native vitreous in both structure and function. It should have similar viscoelastic properties and maintain a normal intraocular pressure (IOP) in order to support the ocular structures in their correct position. It should be optically transparent while allowing the circulation of ions and electrolytes. As a substitute, it should be easy to manipulate and self-renewable in order to require a single implantation. It should also be nontoxic to other ocular structures, biocompatible, nonbiodegradable, readily available at reasonable cost, and easy to store [15]. All vitreous substitutes currently have positive and negative characteristics. New research is underway to discover the ideal vitreous substitute.

## 5. Vitreous Substitutes

There are three major categories of substitute: gases (air, expansile gases), liquids (salt solution, perfluorocarbon liquids, semifluorinated alkanes, silicone oil, etc.), and polymers (hydrogels, smart hydrogels, and thermosetting hydrogels) [15].

### 5.1. Gases

#### 5.1.1. Air

Air present in the vitreous cavity is colourless and inert. It was first used by Ohm in 1911 to repair retinal detachment [15]. Air is inexpensive and easy to find. It remains in the eye for a few days before being replaced by the aqueous humour, thereby reducing its tamponade effect. It is easily absorbed by red blood cells and therefore diffuses quickly into the blood circulation. This is a negative feature of air as a vitreous substitute. Another negative characteristic is its low refractive index (approximately 1.000293 nanometers), which causes complete light reflection and therefore poor optical function [16]. Its use is limited to pneumatic retinopexy at the end of vitrectomy surgery and as an emergency option.

#### 5.1.2. Other Gases

Intraocular gas tamponades have been an important part of vitreoretinal surgery since 1970. Today, sulfur hexafluoride (SF_6_) and perfluoropropane (C_3_F_8_) are increasingly being used in the treatment of many complicated vitreoretinal diseases. Both these gases are heavier than air, colourless, odourless, and nontoxic. They maintain their tamponade effect due to their high surface tension and the diffusion of other gases from the circulation. In 1993, the U.S. Food and Drug Administration approved their use for pneumatic retinopexy [15]. Sulfur hexafluoride expands to double the injected volume within 1 to 2 days and lasts in the vitreous cavity for 1 to 2 weeks. Perfluoropropane expands to about four times its original volume in 72 to 96 hours and lasts for 6 to 8 weeks. For this reason, patients are usually advised to delay air travel and avoid high altitudes for about 2 weeks and 6 weeks following the administration of sulfur hexafluoride and perfluoropropane, respectively.

Due to their buoyancy, intraocular gas tamponades maintain the position of the retina against the RPE, but this effect is limited on the upper part of the bubble and does not affect the inferior retina. For this reason, awkward face-down positioning is required for several days following administration. Adverse effects include an increase of IOP during surgery and, for a few days after injection, gas-induced cataract formation and corneal endothelial changes [17, 18, 19].

### 5.2. Liquids

#### 5.2.1. Perfluorocarbon Liquid (PFCL)

Perfluorocarbon liquid is a fluorochemical in which all the hydrogen atoms are replaced by fluorine [20]. PFCL has a high specific gravity of between 1.76 and 2.03 g/mL with low surface tension and viscosity and an optical transparency. PFCL was initially used in medicine when it was discovered to carry oxygen atoms in the same manner as the blood [21]. In 1987, Chang et al. used PFCL for the first time in retinal detachment with severe proliferative vitreoretinopathy [22, 23]. During vitrectomy, PFCL flattens the detached retina and displaces the subretinal fluid. Furthermore, its transparency facilitates its ease of use during procedures and intraoperative photocoagulation.

The applications of PFCL are, in part, limited to intraoperative use due to its long-term toxicity. This toxicity begins in the inferior retina with mechanical damage to cells via compression and disorganization of the retinal structure and emulsification six days after surgery [24]. In addition, young patients are at a high risk for developing severe ocular inflammation. Recent developments in PFCL have focused on perfluorocarbon-perfused vitrectomy, in which oxygenated or nonoxygenated PCFL is used instead of balanced salt solutions [20, 25, 26].

#### 5.2.2. Semifluorinated Alkanes (SFAs)

Semifluorinated alkanes were identified in 2000 as a new class of compounds with outstanding properties for use in ophthalmology [15]. They have a perfluorocarbon and hydrocarbon segments, and they are soluble in PFCL, hydrocarbons, and silicone oils with a preferred refraction index (1.3). They are physically inert, colourless, and heavier than water (specific gravity of 1.35 g/mL). The lower specific gravity results in less retinal damage than PCFL, and as such SFAs can be used as a temporary endotamponade for periods from 2 to 3 months [27]. Their collateral effects may include cataract, emulsification, and soft epiretinal membrane [27]. Recently, they have been used in a mixture of silicon oil and SFA.

#### 5.2.3. Silicone Oil (SO)

Silicone oil is a liquid polymerized siloxane with organic side chains. It is a hydrophobic polymer with a specific gravity slightly less than water (0.97 g/mL) and a refractive index similar to that of the vitreous [15]. All SO polymers are of commercial interest for their stability, lubricating properties, and as a vitreous substitute, with high surface tension and viscosity, ease of removal, low toxicity, and transparency. For these reasons, they are the only substance currently accepted for long-term vitreous replacement [15, 28]. Due to their buoyancy, SOs have a tamponade force higher at the apex, facilitating the preservation of anatomical integrity. They are used for complicated retinal detachment, when postoperative airplane travel is planned, and in uncooperative patients.

SO is available in several viscosities, but 1000 and 5000 centistokes are used clinically. SO is usually removed after 3 to 6 months once the retina has attached and retinal traction is absent [15].

Although SO is a good vitreous substitute, it has several disadvantages:

1. Tamponade of the inferior retina is difficult due to its low specific gravity.
2. Emulsification in small droplets into the aqueous can cause proliferative vitreoretinopathy, failed retinal detachment, inflammation, secondary glaucoma, and keratopathy [29, 30]. With the advent of microincision vitreoretinal surgery (MIVS), less viscous silicone oils are preferred. They can be easily introduced and removed via small instruments, but they are easier to emulsify. For this reason, new silicone oils with an increased extensional viscosity are under investigation [29].
3. Increased IOP is common after SO implantation. This could be caused by pupillary block glaucoma, overfill of silicone oil, and chronic elevation due to emulsification in the trabecular meshwork and trabeculitis [29].
4. Decreased choroidal thickness three months following SO implantation [31]. This may be caused by the failure of Müller cells to circulate potassium and the subsequent potassium accumulation, retinal degeneration, and inner retinal and choroidal thinning [32].
5. Intracranial migration through the optic nerve to the lamina cribrosa and the optic chiasm with the development of central scotoma. This complication is very rare and usually occurs in patients with optic nerve abnormalities and glaucoma [33].

#### 5.2.4. Heavy Silicone Oil (HSO)

Heavy silicone oil is a tamponade agent formed from a mixture of SO and partially fluorinated octane (PFA) that is heavier than water. For this reason, it has been used for complex retinal detachment involving the inferior part of the retina complicated by proliferative vitreoretinopathy. In 2011, the heavy silicone oil study [34] compared standard SO with HSO in the treatment of inferior retinal detachment. Although superiority of a heavy tamponade was not shown, the study demonstrated a good intraocular tolerance of HSO and no significant emulsification [15].

Complications of HSO include cataract, anterior segment inflammation, emulsification, and elevated IOP [15, 19, 35, 36].

#### 5.2.5. Hydrogels

Cross-linked hydrogels are synthetic polymer networks that are expanded throughout their volume by water. For this reason, they can melt in water without dissolving. Hydrogels have favourable properties such as transparency, biocompatibility, and mechanical flexibility which have led to their widespread application in ophthalmology as soft contact lenses, intraocular lenses, drug delivery systems, and adhesion for wound repair [37].

Hydrogels can be divided into hydrogels and “smart hydrogels.” Smart hydrogels can create a three-dimensional structure in response to a variety of signals including pH variation, temperature, light, pressure, chemicals, and electric fields [38].

To date, these polymers have only been used on an experimental basis. A major disadvantage is their activation of the immune system, resulting in intravitreal inflammation and phagocytosis by macrophages in the vitreous, neuroretina, and subretinal spaces [39]. In addition, they are difficult to sterilize because heat sterilization may cause a degradation of their physical structure. More research is needed to optimize the use of these new molecules as vitreous substitutes.

## 6. Future Vitreous Substitutes

In the recent years, bioengineering studies using rabbit models have shown promising results in a capsular artificial vitreous body made from elastomer rubber with a valve system full of SO or a balanced solution [40]. However, the biocompatibility of a synthetic implant with the human eye is yet untested.

The properties of new hydrogels make them promising candidates as infill biomaterials for the treatment of retinal detachment. Hayashi et al. reported a new class of hydrogel with extremely low swelling pressure which functioned as an artificial vitreous body for over a year without adverse effects in the eyes of rabbits [41].

Another fascinating possibility for the future is the use of cell culture and gene therapy to artificially synthesize the vitreous via the proliferation of hyalocytes [42]. This research has been aided by the use of reverse transcriptase polymerase chain reaction to analyze and compare the expression profiles of several genes involved in synthesis of the vitreous [43].

## 7. Microincision Vitreoretinal Surgery and Vitreous Substitutes

Considerable progress has been recently made in the field of vitreoretinal surgery. One of the most important advancements is the development of small-gauge suturless transconjunctival surgery, also known as minimally invasive vitreous surgery (MIVS). This system uses microcannulas and trocars of 23G (0.64 mm), 25G (0.51 mm) and 27G (0.40 mm), and other instrumentation smaller than traditional 20G (0.9 mm) vitrectomy. This new procedure has introduced many benefits, including a reduction of postoperative inflammation at the sclerotomy site, the need for less tissue manipulation, easier recovery, and a decrease in surgical complications.

Tamponades have begun to adapt with this new type of vitreoretinal surgery. The key feature of vitreous substitutes used in MIVS is a low viscosity, in particular for liquids and notably for SO. A less viscous oil would be user-friendly, but an oil with greater viscosity would be less likely to emulsify [44]. New SO has an increasing extensional viscosity with a greater resistance to emulsification and an improved ease of handling.

In accordance with Poiseuille's law, which evaluates the radius and the length of a tube to calculate the flow of a fluid, MIVS uses special devices including a large syringe, a short infusion line, and a nondistensible material to reduce resistance during the injection and removal of SO [44].

The surgical technique used to inject SO into the eye has also changed with MIVS. There is a diffuse consensus to perform air-silicone exchange instead of fluid-silicone exchange (unused due to the high risk of undesired subretinal silicone drops) or PFCL-silicone exchange (direct exchange). This is preferred to the direct exchange usual for uncomplicated retinal detachment with mild periphery or posterior pole break because it is easier to inject SO using a cannula instead of an infusion line.

The removal of SO with MIVS requires more time than the same procedure using a 20G system, but requires less time positioning and removing trocars.

Surgeons have a choice of many tamponades for use with MIVS, each with advantages over the surgical complications associated with 20G vitrectomy, enabling selection appropriate to the underlying disease [45].

## 8. Conclusion

For many years, it was thought that the vitreous had a marginal role in the anatomy and function of the eye. Only with the advent of new surgical techniques was its importance in maintaining an optimal environment for the retina and the other surrounding tissues recognized.

For this reason, research was originally focused on finding a vitreous substitute with the same physical and biochemical properties of the original vitreous.

In recent years, with the advent of MIVS, it has been necessary to modify the physical structure of the most commonly used vitreous substitutes.

A new generation of vitreous substitutes is under development to satisfy the need for a physiologically equivalent and long-lasting substitute [40, 41, 42, 43].

Future developments based on stem cells and gene therapy may go some way to fulfill the needs of both patients and ophthalmic surgeons.

## References

[1] Grus F. H., Joachim S. C., Pfeiffer N. (2007). Proteomics in ocular fluids. *Proteomics. Clinical Applications*.

[2] Bishop P. N. (2000). Structural macromolecules and supramolecular organisation of the vitreous gel. *Progress in Retinal and Eye Research*.

[3] Ramesh S., Bonshek R. E., Bishop P. N. (2004). Immunolocalisation of opticin in the human eye. *The British Journal of Ophthalmology*.

[4] Castoro J. A., Bettelheim F. A. (1986). Topographic distribution of water in rhesus monkey vitreus. *Ophthalmic Research*.

[5] Wong R. W., Richa D. C., Hahn P., Green W. R., Dunaief J. L. (2007). Iron toxicity as a potential factor in AMD. *Retina*.

[6] Bishop P. N., Crossman M. V., McLeod D., Ayad S. (1994). Extraction and characterization of the tissue forms of collagen types II and IX from bovine vitreous. *The Biochemical Journal*.

[7] Ponsioen T. L., Luyn M. J., Worp R. J., Pas H. H., Hooymans J. M., Los L. I. (2008). Human retinal muller cells synthesize collagens of the vitreous and vitreoretinal interface in vitro. *Molecular Vision*.

[8] Lumi X., Hawlina M., Glavac D. (2015). Ageing of the vitreous: from acute onset floaters and flashes to retinal detachment. *Ageing Research Reviews*.

[9] Theocharis D. A., Skandalis S. S., Noulas A. V., Papageorgakopoulou N., Theocharis A. D., Karamanos N. K. (2008). Hyaluronan and chondroitin sulfate proteoglycans in the supramolecular organization of the mammalian vitreous body. *Connective Tissue Research*.

[10] Buzza M. S., Zamurs L., Sun J. (2005). Extracellular matrix remodeling by human granzyme B via cleavage of vitreonectin, fibronectin and laminin. *The Journal of Biological Chemistry*.

[11] DiMattio J. (1989). A comparative study of ascorbic acid entry into aqueous and vitreous humors of the rat and guinea pig. *Investigative Ophthalmology & Visual Science*.

[12] Shui Y. B., Holekamp N. M., Kramer B. C. (2009). The gel state of the vitreous and ascorbate-dependent oxygen consumption: relationship to the etiology of nuclear cataracts. *Archives of Ophthalmology*.

[13] Miyamoto T., Inoue H., Sakamoto Y. (2005). Identification of a novel splice site mutation of the CSPG2 gene in a Japanese family with Wagner syndrome. *Investigative Ophthalmology & Visual Science*.

[14] Boettner E. A., Wolter J. R. (1962). Transmission of the ocular media. *Investigative Ophthalmology*.

[15] Kleinberg T. T., Tzekov R. T., Stein L., Ravi N., Kaushal S. (2011). Vitreous substitutes: a comprehensive review. *Survey of Ophthalmology*.

[16] Donati S., Caprani S. M., Airaghi G. (2014). Vitreous substitutes: the present and the future. *BioMed Research International*.

[17] Lee D. A., Wilson M. R., Yoshizumi M. O., Hall M. (1991). The ocular effects of gases when injected into the anterior chamber of rabbit eyes. *Archives of Ophthalmology*.

[18] Wilkinson C., Rice T., Craven L. (1996). Instrumentation, materials, and treatment alternatives. *Michels Retinal Detachment*.

[19] Vaziri K., Schwartz S. G., Kishor K. S., Flynn H. W. (2016). Tamponade in the surgical management of retinal detachment. *Clinical Ophthalmology*.

[20] Yu Q., Liu K., Su L., Xia X., Xu X. (2014). Perfluorocarbon liquid: its application in vitreoretinal surgery and related ocular inflammation. *BioMed Research International*.

[21] Shaffer T. H., Wolfon M. R., Clark L. C. (1992). Liquid ventilation. *Pedriatic Pulmonology*.

[22] Chang S., Ozmert E., Zimmerman N. J. (1988). Intraoperative liquids in the management of proliferative vitreoretinopathy. *American Journal of Ophthalmology*.

[23] Georgalas I., Ladas I., Tservakis I. (2011). Perfluorocarbon liquids in vitreoretinal surgery: a review of applications and toxicity. *Cutaneous and Ocular Toxicology*.

[24] Matteucci A., Formisano G., Paradisi S. (2007). Biocompatibility assessment of liquid artificial vitreous replacements: relevance of in vitro studies. *Survey of Ophthalmology*.

[25] Arevalo J. F. (2008). En bloc perfluorodissection in vitreoretinal surgery: a new surgical technique. *Retina*.

[26] Wagenfeld O., Zeitz O., Skevas C., Richard G. (2010). Long-lasting endotamponades in vitreoretinal surgery. *Ophthalmologica*.

[27] Kirchhof B., Wong D., Meurs J. (2002). Use of perfluorohexyloctane as a long-term internal tamponade agent in complicated retinal detachment surgery. *American Journal of Ophthalmology*.

[28] Kim R. W., Baumal C. (2004). Anterior segment complications related to vitreous substitutes. *Ophthalmology Clinics of North America*.

[29] Barca F., Caporossi T., Rizzo S. (2014). Silicone oil: different physical proprieties and clinical applications. *BioMed Research International*.

[30] Kawaguchi M. (2016). Silicone oil emulsions stabilized by polymers and solid particles. *Advances in Colloid and Interface Science*.

[31] Odrobina D., Golebiewska J., Maroszynska I. (2016). Choroidal thickness changes after vitrectomy with silicone oil tamponade for proliferative vitreoretinopathy retinal detachment. *Retina*.

[32] Winter M., Eberhardt W., Scholz C., Reichenbach A. (2000). Failure of potassium siphoning by Muller cells: a new hypothesis of perfluorocarbon liquid-induced retinopathy. *Investigative Ophthalmology & Visual Science*.

[33] Grzybowski A., Pieczynski J., Ascaso F. J. (2014). Neuronal complications of intravitreal silicone oil: an updated review. *Acta Ophthalmologica*.

[34] Joussen A. M., Rizzo S., Kirchhof B. (2011). Heavy silicone oil versus standard silicone oil in as vitreous tamponade in inferior PVR (HSO Study): interim analysis. *Acta Ophthalmologica*.

[35] Morescalchi F., Costagliola C., Duse S. (2014). Heavy silicone oil and intraocular inflammation. *BioMed Research International*.

[36] Schwartz S. G., Flynn H. W., Lee W. H., Wang X. (2014). Tamponade in surgery for retinal detachment associated with proliferative vitreoretinopathy. *Cochrane Database of Systematic Reviews*.

[37] Kirchhof S., Goepferich A. M., Brandl F. P. (2015). Hydrogels in ophthalmic applications. *European Journal of Pharmaceutics and Biopharmaceutics*.

[38] Chaterji S., Kwon I. K., Park K. (2007). Smart polymeric gels: redefining the limits of biomedical devices. *Progress in Polymer Science*.

[39] Swindle-Reilly K. E., Shah M., Hamilton P. D., Eskin T. A., Kaushal S., Ravi N. (2009). Rabbit study of an in situ forming hydrogel vitreous substitute. *Investigative Ophthalmology & Visual Science*.

[40] Gao Q., Mou S., Ge J. (2008). A new strategy to replace the natural vitreous by a novel capsular artificial vitreous body with pressure-control valve. *Eye*.

[41] Hayashi K., Okamoto F., Hoshi S. (2017). Fast-forming hydrogel with ultralow polymeric content as an artificial vitreous body. *Nature Biomedical Engineering*.

[42] Sommer F., Kobuch K., Brandl F. (2007). Ascorbic acid modulates proliferation and extracellular matrix accumulation of hyalocytes. *Tissue Engineering*.

[43] Kashiwagi Y., Nishitsuka K., Takamura T., Yamamoto T., Yamashita H. (2011). Cloning and characterization of human vitreous tissue-derived cells. *Acta Ophthalmologica*.

[44] Rizzo S., Barca F. (2014). Vitreous substitute and tamponade substances for microincision vitreoretinal surgery. *Developments in Ophthalmology*.

[45] Grosso A., Charrier L., Lovato E. (2014). Twenty-five-gauge vitrectomy versus 23-gauge vitrectomy in the management of macular diseases: a comparative analysis through a health technology assessment model. *International Ophthalmology*.

